# Potential of GSPT1 as a novel target for glioblastoma therapy

**DOI:** 10.1038/s41419-024-06967-1

**Published:** 2024-08-08

**Authors:** Takashi Sasayama, Takeshi Hamada, Kazuhiro Tanaka, Hiroaki Nagashima, Shunsuke Yamanishi, Takehiko Ueyama

**Affiliations:** 1 Department of Neurosurgery, Kobe University Graduate School of Medicine, Kobe, Japan; 2 Laboratory of Molecular Pharmacology, Biosignal Research Center, Kobe University, Kobe, Japan

**Keywords:** Cancer in the nervous system, CNS cancer

## Abstract

Glioblastoma is the most common malignant brain tumor in adults, the survival rate of which has not significantly improved over the past three decades. Therefore, there is an urgent need to develop novel treatment modalities. We previously reported that G1 to S phase transition 1 (GSPT1) depletion induces delayed cell cycle in primary astrocytes. Herein, we examined the potential of GSPT1 as a novel target for glioblastoma therapy. CC-885, a cereblon modulator that degrades GSPT1 by bridging GSPT1 to the CRL4 E3 ubiquitin ligase complex, was administered to nude mice with transplanted brain tumors of U87 glioblastoma cells. The survival period was significantly longer in CC-885 treated mice than in control mice. Furthermore, we generated GSPT1-knockout (KO) U87 cells and GSPT1-KO U87 cells with stable overexpression of FLAG-tagged GSPT1 (Rescued GSPT1-KO). Mice with transplanted GSPT1-KO U87 cells and Rescued GSPT1-KO U87 cells showed significantly longer and similar survival periods, respectively, as those with wild-type (WT) U87 cells. GSPT1-KO U87 cells showed enhanced apoptosis, detected by cleaved PARP1, compared to WT U87 cells. Brain tumors with transplantation of GSPT1-KO U87 cells also showed enhanced apoptosis compared to those with transplantation of WT and Rescued GSPT1-KO U87 cells. GSPT1 expression was confirmed in patients with glioblastoma. However, the clinical study using 87 glioblastoma samples showed that GSPT1 mRNA levels were not associated with overall survival. Taken together, we propose that GSPT1 is an essential protein for glioblastoma growth, but not its malignant characteristics, and that GSPT1 is a potential target for developing glioblastoma therapeutics.

## Introduction

Astrocytoma/glioma, classified into grades I–IV on the basis of histological and genomic characteristics, is one of the most common brain tumors in adults. Glioblastoma (also called glioblastoma multiform or grade IV astrocytoma) is the most common malignant brain tumor, accounting for more than 50% of all primary brain tumors in adults, with a median survival of 14–20 months after treatment with temozolomide (TMZ), vascular endothelial growth factor (VEGF) inhibitors, radiation, and other adjuvant therapies [1–3]. Due to its remarkable proliferative and invasive capabilities, its survival rate is one of the worst among various cancers [3]. Therefore, developing a novel treatment is urgently needed.

G1 to S phase transition 1 (GSPT1)/eukaryotic releasing factor 3 (eRF3), a GTP-binding protein, was first identified as a molecule involved in G1 to S phase transition in *Saccharomyces cerevisiae* [4]. Subsequently, it was reported that GSPT1 mediates translation termination via the eRF1/eRF3 complex in eukaryotes in a GTP-dependent manner [5]. We previously reported that GSPT1 is a novel downstream target of RAC1 [6], which is a member of the Rho-family small GTPases and has been reported as a tumor-driving and -promoting molecule [7], and that depletion of GSPT1 and RAC1 induced delayed proliferation of glioblastoma (LN229) cells and primary astrocytes [6]. Furthermore, we demonstrated that RAC1, and not GSPT1, promoted cell migration in primary astrocytes. These results suggest that depletion/deletion of GSPT1 is a novel therapeutic target for astrocyte proliferation-associated diseases, such as astrocytoma, and injury, infarction, and inflammation in the central nervous system.

Thalidomide was approved for the treatment of multiple myeloma in the 1990s; currently, thalidomide derivatives, including lenalidomide and pomalidomide, are being used for the treatment of multiple myeloma [8]. CC-885, a thalidomide derivative, is an immunomodulatory drug that binds to cereblon (CRBN) and leads to GSPT1 degradation [9]. CRBN is a substrate-receptor component of the E3 ubiquitin-ligase complex containing cullin 4 (Cul4), known as the Cul4-really interacting new gene (RING) ligase complex (CRL4). The CRL4 E3 ubiquitin ligase complex consists of a core scaffolding component, Cul4, which binds to the RING finger protein regulator of cullins (ROC1), the docking site for E2 recruitment, and DNA damage-binding protein 1 (DDB1), an adaptor protein (Cul4-Roc1-DDB1). DDB1 provides a binding site for CRL4 substrate-receptor proteins, such as CRBN, which recruits substrates to the CRL4 E3 ubiquitin ligase complex and determines its substrate specificity (CRL4^CRBN^), leading to its polyubiquitination and subsequent proteasomal degradation [9, 10]. CRBN modulators, which bind to CRBN and alter its substrate specificity, enable CRL4^CRBN^ to degrade neosubstrates [11]. This strategy of using CRBN modulators as “molecular glues” that mediate the interaction between CRBN and neosubstrates is applied to the treatment of hematologic malignancies. However, the effects of GSPT1 depletion/deletion and thalidomide derivatives on primary brain tumors have not yet been reported.

Herein, to examine the potency of GSPT1 as a novel target for primary brain tumor treatment, we used CC-885 to treat brain tumors produced by transplantation of U87 glioblastoma cells into the brains of nude mice. Additionally, we generated a GSPT1 knockout (GSPT1-KO) U87 glioblastoma cell line by genome editing along with a rescued GSPT1-KO U87 glioblastoma cell line with stable overexpression of FLAG-tagged GSPT1 (Rescued GSPT1-KO) and examined the effects of GSPT1 using these cells and the transplantation brain tumor model. Furthermore, we examined GSPT1 expression levels and the relationship between GSPT1 mRNA levels and patient prognosis using glioblastoma samples from 87 patients.

## Results

### Overexpression of EGFP-GSPT1 rescued cell-cycle delay induced by RAC1-KO in primary astrocytes

**Fig. 1:**
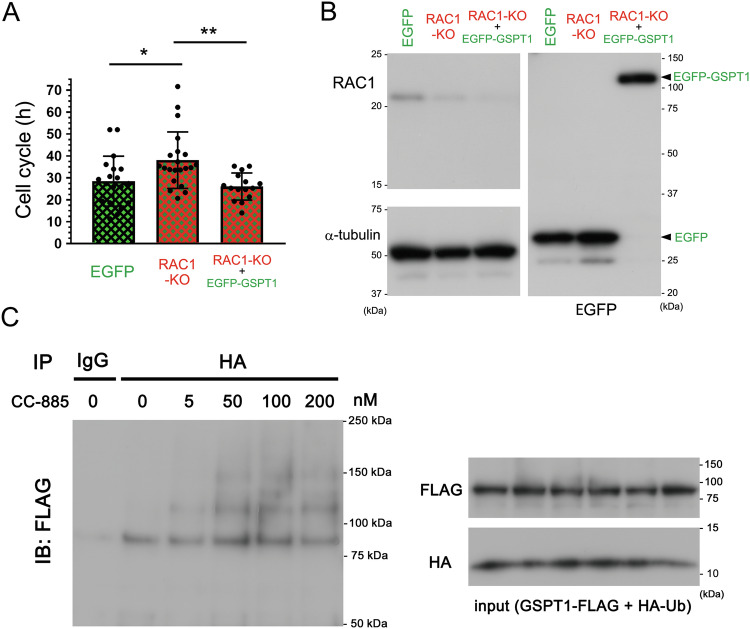
Rescued cell-cycle delay of RAC1-KO astrocytes by GSPT1 overexpression and enhanced GSPT1 ubiquitination by CC-885 in U87 cells. **A** WT primary astrocytes with EGFP overexpression, RAC1-KO (with tdTomato expression) with EGFP overexpression, and RAC1-KO with EGFP-GSPT1 overexpression were prepared by electroporation. Cell cycles were evaluated using a live imaging LCV110 system and plotted. *n* = 19 [EGFP (control)], 21 (RAC1-KO + EGFP), and 20 (RAC1-KO + EGFP-GSPT1). *P = 0.0213 and **P = 0.0055 by one-way ANOVA with Tukey’s post-hoc test. **B** RAC1 and EGFP expression in WT primary astrocytes along with RAC1-KO and EGFP or EGFP-GSPT1 expression in RAC1-KO primary astrocytes were confirmed by immunoblotting using RAC1 and EGFP antibodies, respectively. Comparative loading of proteins was confirmed by immunoblotting using α-tubulin antibody. **C** GSPT1-FLAG and HA-Ub plasmids were co-transfected into U87 glioblastoma cells by lipofection. Thirty hours after transfection, cells were treated with the indicated concentrations of CC-885 for 16 h. Cell lysates were immunoprecipitated using HA antibody, and then immunoblotted using FLAG antibody. Comparative expression and loading of proteins were confirmed by immunoblotting using ΗΑ and FLAG antibodies.

We previously demonstrated that depletion of GSPT1, a downstream target molecule of RAC1, and deletion of RAC1 in primary astrocytes resulted in delayed cell-cycle progression [6]. In the present study, we showed that the delayed cell cycle in primary astrocytes obtained from astrocyte-specific RAC1-KO (*GFAP-Cre;RAC1*^*flox/flox*^*;tdTomato*) mice was completely rescued by overexpression of EGFP-tagged GSPT1 (EGFP-GSPT1) in comparison to wild-type (WT) primary astrocytes (Fig. 1A, B), confirming that GSPT1 is the main downstream target of RAC1-involved cell cycle signaling in astrocytes.

### CC-885 promoted ubiquitination of GSPT1 in U87 glioblastoma cells

We previously reported that HeLa cells, a cell line derived from cancer of the uterine cervix, demonstrated high GSPT1 expression [6]. In the current study, we found that U87 glioblastoma cells demonstrated higher GSPT1 expression than LN229 glioblastoma cells and that U87 cells showed similar expression of GSPT1 as HeLa cells (Fig. S1A). Overexpression of GSPT1-EGFP showed cytoplasmic localization in HeLa cells (Fig. S1B). In U87 cells, overexpression of non-tagged and endogenous GSPT1 also showed cytoplasmic localization (Fig. S1C). CC-885 reportedly degrades GSPT1 through polyubiquitination via the E3 ubiquitin ligase complex (CRL4^CBRN^) [9]. Herein, we confirmed the ubiquitination effect of CC-885 on GSPT1 in U87 cells overexpressing GSPT1-FLAG and HA-Ub. GSPT1-FLAG was ubiquitinated by CC-885 in a dose-dependent manner and peaked at 100 nM (Fig. 1C).

### CC-885 inhibited growth of transplanted U87 brain tumor and improved survival rate

**Fig. 2:**
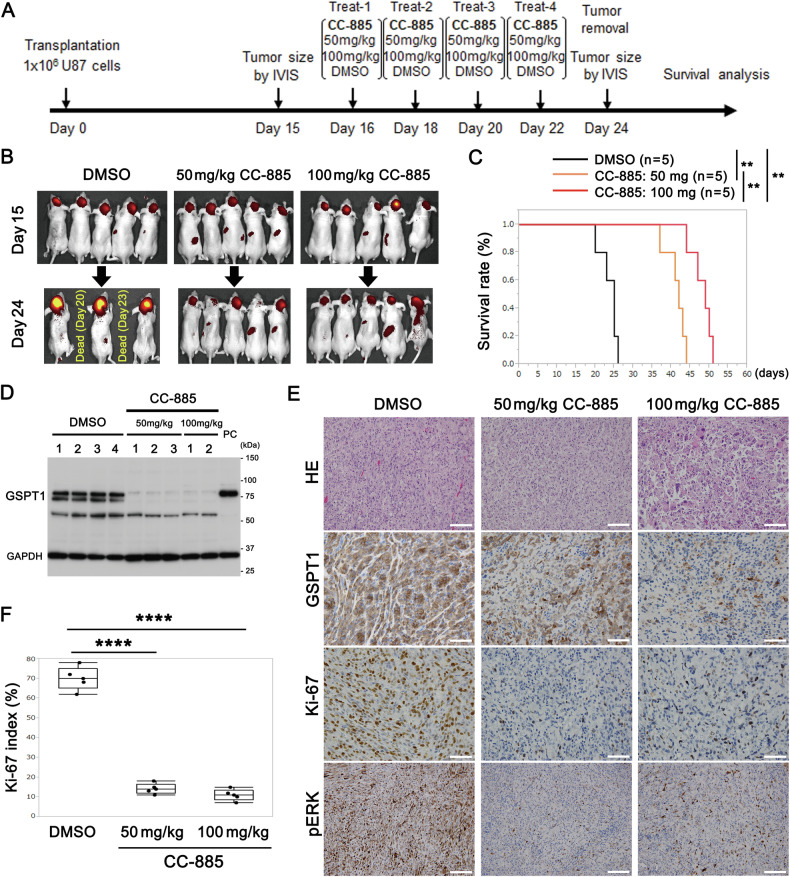
Prolonged survival of nude mice with transplanted U87 cells by CC-885 treatment. 1 × 10^6^ U87 glioblastoma cells expressing iRFP720 were transplanted into the brains of nude mice. 50 or 100 mg/kg of CC-885 dissolved in DMSO and the same volume of DMSO (as control) was intraperitoneally administered to nude mice on days 16, 18, 20, and 22 (4 times in total). **A** Chart of transplantation, CC-885 administration, and tumor size evaluation using an in vivo imaging system (IVIS). **B** Tumor size was assessed using the IVIS on days 15 and 24. **C** Survival period from transplantation to death (*x*-axis) and survival rate (*y*-axis) were plotted. *n* = 5. **P = 0.0021 (DMSO vs. 50 mg/kg CC-885), **P = 0.0021 (DMSO vs. 100 mg/kg CC-885), and **P = 0.0044 (50 vs. 100 mg/kg CC-885) by the Kaplan–Meier method and log-rank test. **D** Effects of CC885 on GSPT1 expression levels in brain tumors were evaluated by immunoblotting [*n* = 3 (50 mg/kg CC-885), 2 (100 mg/kg CC-885), and 4 (DMSO)] using GSPT1 antibody on day 24. Comparative loading of proteins was confirmed by immunoblotting with GAPDH antibody. **E** Effects of CC-885 on brain tumors were evaluated by immunostaining. The transplanted brain tumors (WT, WT with CC-885 treatment) were removed, fixed, and embedded in paraffin on day 24. The samples were prepared for hematoxylin-eosin (HE) staining and immunostaining (with hematoxylin counterstaining) using GSPT1, Ki-67, and phosphor-ERK1/2 antibodies. *n* = 5; scale bars: 100 μm (HE, GSPT1, and pERK) and 50 μm (Ki-67). **F** Ki67 immunostaining indices in the brain tumors were measured using ImageJ software, and the Ki67 index was plotted. ****P < 0.0001 by one-way ANOVA with Tukey’s post hoc test.

To examine the effect of CC-885 on brain tumors, U87 glioblastoma cells expressing iRFP720 were transplanted into the brains of nude mice. Three experimental groups were formed: control (DMSO), 50 mg/kg CC-885, and 100 mg/kg CC-885 (Fig. 2A). The experimental protocol is shown in Fig. 2A. Tumor size was evaluated using an IVIS Lumina LT on days 15 (before CC-885 treatment) and 24 (after CC-885 treatment) after transplantation, and CC-885 was administered intraperitoneally on days 16, 18, 20, and 22 (a total of 4 times every alternate day). In the control group, 2 of 5 mice died on days 20 and 23. The iRFP720 fluorescence intensity of the transplanted brain tumors in the 50 and 100 mg/kg CC-885 groups was lower than that in the control group (Fig. 2B). The survival periods were significantly prolonged in both 50 and 100 mg/kg CC-885 groups compared to that in the control group (Fig. 2C). Decreased expression of GSPT1 in CC-885 treated groups was confirmed by immunoblotting (Fig. 2D) and immunostaining (Fig. 2E) using dissected transplanted brain tumors on day 24. Hematoxylin-eosin (HE) staining showed that tumor cells were significantly swollen, some were multinucleated, and that increased cell polymorphism was observed due to CC-885 treatment in comparison to the control group (Fig. 2E). Furthermore, immunostaining of Ki-67 and phosphorylated ERK1/2, which are indicators of cell proliferation, significantly decreased in the CC-885 treated groups compared to the control group (Fig. 2E, F). These results suggest that decreased GSPT1 levels delayed the growth of transplanted brain tumors and prolonged the survival period in mice.

### Delayed growth and prolonged survival by GSPT1-KO

**Fig. 3:**
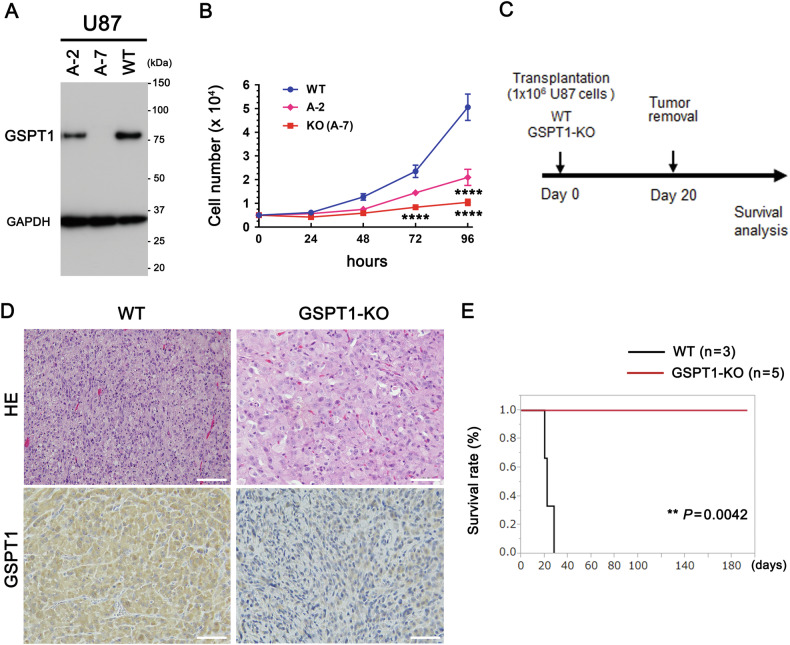
Prolonged survival of nude mice transplanted with GSPT1-KO U87 cells. **A** GSPT1-KO U87 glioblastoma cells were generated by genome editing. Heterozygous (A-2) and homozygous (A-7) GSPT1-KO U87 cells were confirmed by immunoblotting with GSPT1 antibody. Comparative loading of proteins was confirmed by immunoblotting using GAPDH antibody. **B** Growth rates of the three cell lines were evaluated and plotted. *n* = 19 (WT), 4 (A-2), and 14 [KO (A-7)]. ****P < 0.0001 between WT and KO (A-7) (at 72 and 96 h) and WT and A-2 (96 h) by two-way ANOVA with Tukey’s post-hoc test. No significant differences were observed between A-2 vs A-7. **C** Experimental chart: 1 × 10^6^ WT and GSPT1-KO (A-7) U87 cells were transplanted into the brains of nude mice. **D** Brain tumors transplanted with WT or GSPT1-KO U87 cells were removed, fixed, embedded in paraffin on day 20, stained with hematoxylin and eosin (HE), and immunostained for GSPT1 (*n* = 3). Scale bars, 100 μm. **E** The survival period from transplantation to death (*x*-axis) and survival rate (*y*-axis) were plotted. *n* = 3 (WT) and 5 (GSPT1-KO). **P = 0.0042 by the Kaplan–Meier method and log-rank test.

To confirm the relationship between GSPT1 expression levels and the survival period, we generated GSPT1-KO U87 glioblastoma cells using genome editing. Heterozygous GSPT1-KO (A-2) and homozygous GSPT1-KO (A-7) clones were obtained and confirmed by immunoblotting (Fig. 3A). The growth rate of the A-7 clone (hereafter referred to as GSPT1-KO) was significantly slower than that of WT cells 72 h after dissemination (Fig. 3B). In contrast, the growth rate of the A-2 clone was significantly slower than that of WT cells after 96 h (Fig. 3B). Next, GSPT1-KO and WT U87 cells were transplanted into the brains of nude mice (Fig. 3C). Lower expression levels of GSPT1 in the brain tumors transplanted with GSPT1-KO U87 cells than in those transplanted with WT U87 cells were confirmed by immunostaining on day 20 (Fig. 3D). HE staining showed that tumor cells in the brain tumors transplanted with GSPT1-KO U87 cells were swollen and showed pleomorphic nuclei (Fig. 3D). The survival period of mice transplanted with GSPT1-KO cells was significantly prolonged compared to that of mice transplanted with WT cells (Fig. 3E).

### Cancelled phenotypes (delayed growth and prolonged survival) of GSPT1-KO by rescued GSPT1 overexpression

**Fig. 4:**
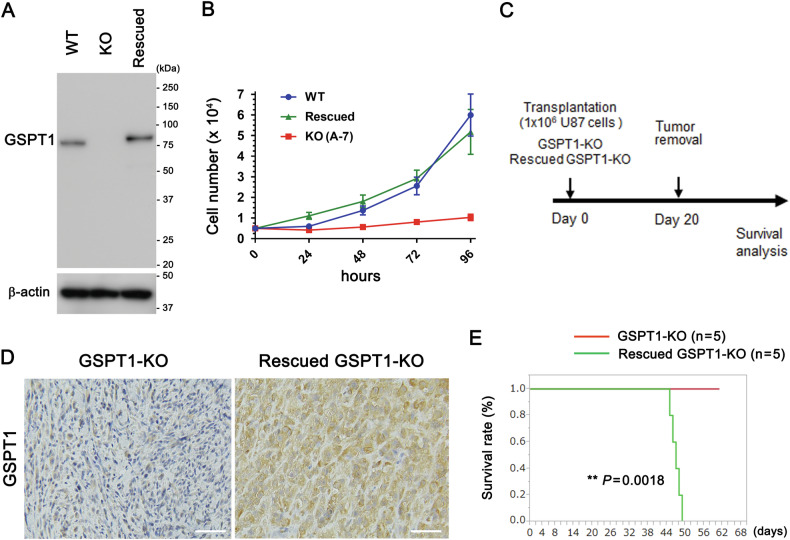
Cancelled prolonged survival of nude mice transplanted with Rescued GSPT1-KO U87 cells. **A** GSPT1-KO (A-7) with stable overexpression of GSPT1-FLAG (Rescued GSPT1-KO) U87 glioblastoma cells were generated. Rescued and comparable expression of GSPT1-FLAG in Rescued GSPT1 U87 cells, compared to that in WT U87 cells, was confirmed by immunoblotting using GSPT1 antibody. Comparative loading of proteins was confirmed by immunoblotting using β-actin antibody. **B** Growth rates of the three cell lines were evaluated and plotted (*n* = 10 in WT and Rescued GSPT1-KO). No significant difference was observed between WT and Rescued GSPT1-KO U87 cells by two-way ANOVA with Tukey’s post-hoc test. Growth curve of GSPT1-KO(A-7) cells is shown as a reference. **C** Experimental chart: 1 × 10^6^ GSPT1-KO as well as Rescued GSPT1-KO U87 cells were transplanted into the brains of nude mice. **D** Brain tumors transplanted with GSPT1-KO or Rescued GSPT1-KO U87 cells were removed, fixed, embedded in paraffin on day 20, and immunostained for GSPT1 (*n* = 5). Scale bars: 100 μm. **E** Survival period from transplantation to death (*x*-axis) and survival rate (*y*-axis) were plotted. *n* = 5; **P = 0.0018 by the Kaplan–Meier method and log-rank test.

To further confirm the effects of GSPT1 on cell growth and survival of mice, we generated GSPT1-KO U87 glioblastoma cells with stable overexpression of GSPT1-FLAG (hereafter referred to as Rescued GSPT1-KO U87 cells). Comparable expression levels of GSPT1-FLAG in the Rescued GSPT1-KO U87 cells were confirmed by immunoblotting compared to that in the WT U87 cells (Fig. 4A). The growth rate of Rescued GSPT1-KO U87 cells was similar to that of WT U87 cells (Fig. 4B). Next, GSPT1-KO and Rescued GSPT1-KO U87 cells were transplanted into the brains of nude mice (Fig. 4C). Higher expression levels of GSPT1 in the brain tumors transplanted with Rescued GSPT1-KO U87 cells than in those transplanted with GSPT1-KO U87 cells were confirmed by immunostaining on day 20 (Fig. 4D). The survival period of mice transplanted with Rescued GSPT1-KO U87 cells was significantly shorter than that of mice transplanted with GSPT1-KO U87 cells (Fig. 4E).

### Enhanced apoptosis in GSPT1-KO U87 cells and brain tumors transplanted with GSPT1-KO U87 cells

**Fig. 5:**
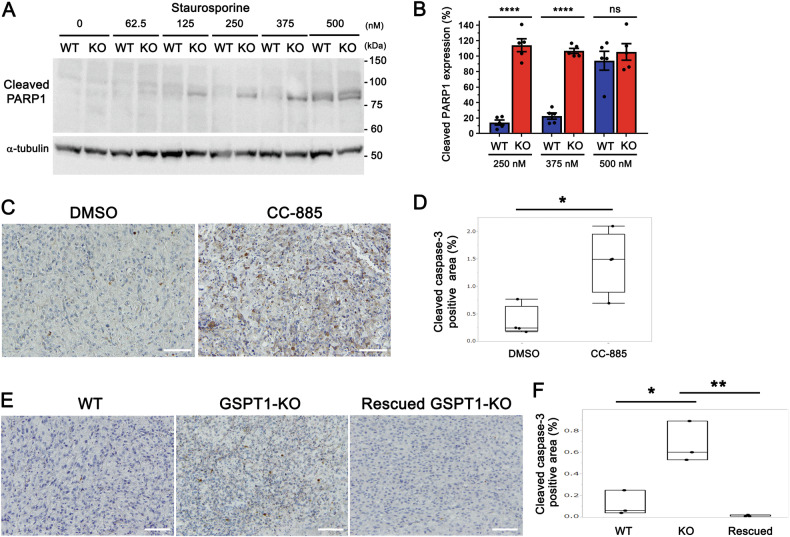
Vulnerability of GSPT1-KO U87 cells to apoptosis. **A**, **B**, WT and GSPT1-KO U87 glioblastoma cells were treated with the indicated concentrations (62.5–500 nM) of staurosporine for 9 h. To evaluate apoptosis, WT and GSPT1-KO U87 cell lysates were subjected to immunoblotting using cleaved PARP1 antibody (**A**). Cleaved fragments of PARP1 were normalized by α-tubulin at each concentration (250, 375, and 500 nM) of staurosporine and plotted (**B**). *n* = 5; ****P < 0.0001 by Student *t*-test; ns non-significant. **C**–**F**, Brain tumors transplanted with WT U87 cells or WT U87 cells treated with CC-885 (**C**, day 24) and WT, GSPT1-KO, or Rescued GSPT1-KO U87 cells (**E**, day 20) were removed, fixed, and embedded in paraffin. The samples were prepared for immunostaining using cleaved caspase-3 (scale bars: 100 μm). Cleaved caspase-3-positive areas were measured using ImageJ software and plotted. **D** *P = 0.0142 by Student *t*-test. *n* = 4. **F** *P = 0.0125 (WT vs. GSPT1-KO) and **P = 0.0039 (GSPT1-KO vs. Rescued GSPT1-KO) by one-way ANOVA with Tukey’s post-hoc test. No significant differences were observed between WT and Rescued GSPT1-KO. *n* = 3.

To examine the potential of GSPT1 as a therapeutic target for glioblastoma, we evaluated apoptosis in both GSPT1-KO U87 cells and brain tumors produced by transplanted GSPT1-KO U87 cells. First, we treated U87 cells with the well-known apoptosis-inducing drug, staurosporine, and evaluated the levels of the conventionally used apoptosis marker, cleaved PARP1, by immunoblotting. Cleaved PARP1 band levels were stronger in GSPT1-KO U87 cells (up to 375 nM, but not at 500 nM) than in WT U87 cells (Fig. 5A, B). Next, we examined the immunostaining levels of cleaved caspase-3 and cleaved PARP1 in brain tumors transplanted with U87 cells on day 24. The relative value of cleaved caspase 3 positive area was significantly higher in the CC-885 treated group than in the control group (Fig. 5D). Similar results were obtained for cleaved PARP1 (Fig. S2). Furthermore, the relative value of cleaved caspase-3 positive area in brain tumors transplanted with GSPT1-KO U87 cells was higher than that in those transplanted with WT U87 cells and Rescued GSPT1-KO U87 cells on day 20 (Fig. 5E). These results suggest that depletion/deletion of GSPT1 makes glioblastoma cells more susceptible to death than WT U87 cells.

### GSPT1 expression in the glioblastoma samples of patients and relationship between GSPT1 mRNA expression and patient prognosis

**Fig. 6:**
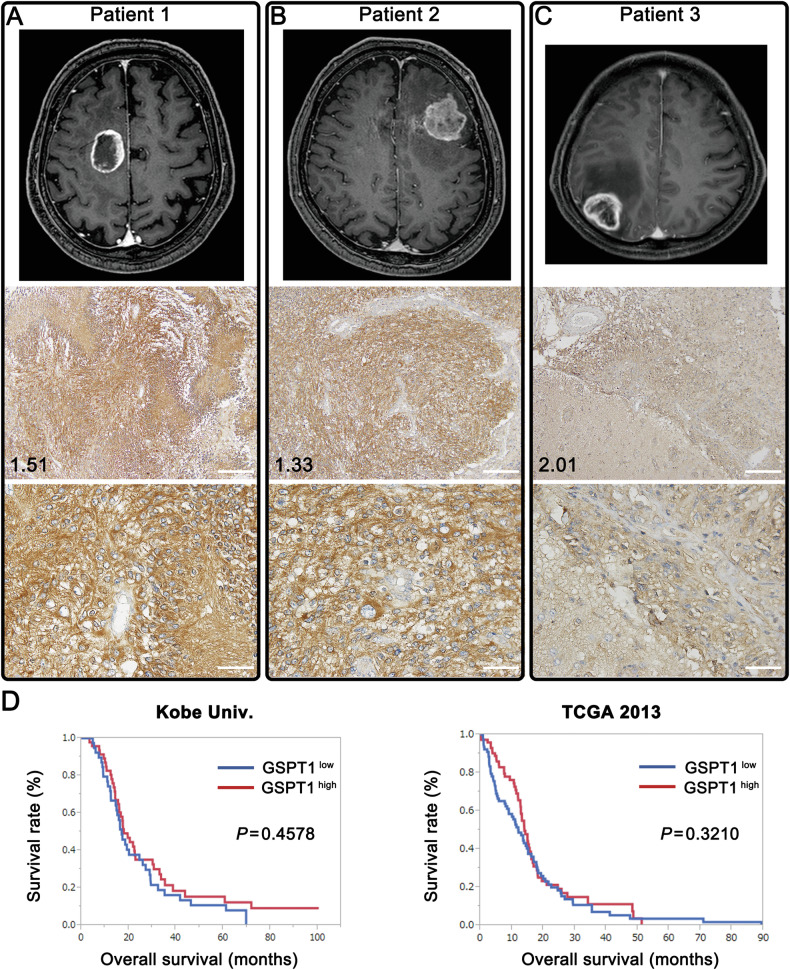
GSPT1 expression in glioblastoma samples of patients, and the relationship between expression levels and patient prognosis. **A**–**C** GSPT1 protein expression in three glioblastoma samples (Patient-1, Patient-2, and Patient-3) from 18 patients (data of the other 15 patients are presented in Fig. S3). Upper panels: gadolinium-enhanced MR images. Middle and lower panels show microphotographs of GSPT1 immunostaining with hematoxylin counterstaining. Patient-1 was a 60-year-old man with a right medial frontal lobe tumor. Patient-2 was a 64-year-old man with a tumor in the left frontal lobe. Patient-3 was a 59-year-old man with a right parietal lobe tumor. GSPT1 is expressed in the cytoplasm of glioblastoma cells. Notably, GSPT1 is not expressed in vascular cells. Patients-1 and -2 showed strong GSPT1 expression; however, Patient-3 showed weak to moderate GSPT1 expression. The numbers at the bottom left of each microphotograph represent the relative expression values of GSPT1 mRNA in real-time RT-PCR analyses. There was no association between GSPT1 immunostaining levels and relative mRNA expression values. Scale bars, 100 μm (upper panels) and 50 μm (lower panels). **D** Comparison of Kaplan–Meier curves of patients with glioblastoma according to the mRNA expression levels of GSPT1 in glioblastoma samples. Eighty-seven patients with glioblastoma were divided into two groups based on the median value of GSPT1 expression. GSPT1^high^: GSPT1 expression values were higher than the median values. GSPT1^low^: GSPT1 expression values were lower than the median values. Left graph: Dataset from Kobe University Hospital. Right graph: Dataset from TCGA Glioblastoma 2013 (https://www.cbioportal.org/), which are freely available.

We examined the expression levels of GSPT1 in glioblastoma samples. As shown in Fig. 6A–C and Fig. S3, GSPT1 protein expression varied (strongly to moderately/weakly) across patient samples. GSPT1 was expressed in the cytoplasm, similar to U87 glioblastoma cells. On the other hand, GSPT1 immunostaining was not observed in the vascular tissue. Next, the relationship between GSPT1 expression and overall survival was examined in patients with glioblastoma. We measured the mRNA expression levels of 87 human glioblastoma specimens using real-time reverse transcription polymerase chain reaction (RT-PCR) and divided them into two groups: a group with values higher than the median (GSPT1^high^) and a group with values lower than the median (GSPT1^low^) (Fig. S4). There was no association between GSPT1 immunostaining levels and relative mRNA expression (Figs. 6A–C and S3, S4). GSPT1 mRNA expression levels did not significantly correlate with the overall survival period in patients with glioblastoma (Fig. 6D left). Additionally, we analyzed the relationship between GSPT1 mRNA expression and prognosis using The Cancer Genome Atlas (TCGA) dataset of Glioblastoma 2013 (https://www.cbioportal.org/), which are freely available for researchers, and found no relationship (Fig. 6D right). These findings suggest that GSPT1 may be an essential protein required for the growth of glioblastoma cells; however, it may not be associated with the malignant characteristics of the tumor.

## Discussion

Matyskiela et al. reported that targeted degradation of GSPT1 through the CC-885-mediated interbridge between GSPT1 and the CRL4^CRBN^ ubiquitin ligase complex shows broad antitumor activity in both cell lines and patient samples of acute myeloid leukemia [9]. These authors also reported that the growth inhibitory IC_50_ values of CC-885 to 6 glioblastoma cell lines were ~5–200 nM [9], consistent with our data shown in Fig. 1C. More recently, GSPT1 was delineated as a tumor-promoting factor in several types of cancers, such as astrocytoma [12], liver cancer [13], gastrointestinal cancer [14], lung cancer [15], and colon cancer [16]. However, the effects of CC-885 on tumors in the central nervous system have not yet been evaluated at an individual level.

In the present study, we demonstrated that CC-885 was effective in treating brain tumors after transplantation of U87 glioblastoma cells in mice. A molecular weight of less than 400~500, along with high lipid solubility, is an important factor for effectively passing the blood-brain barrier [17]. The molecular weight of CC-885 is 440.88. Therefore, thalidomide derivatives/immunomodulatory drugs with <450 molecular weight, which bind to CRBN and lead to degradation of GSPT1, are potentially effective therapeutics for primary brain tumors. The effect of thalidomide derivatives reportedly depends on their binding specificity and affinity to CRBN, as well as CRBN expression levels [18, 19]. Indeed, CRBN is expressed at medium levels in gliomas (https://www.proteinatlas.org/ENSG00000113851-CRBN/pathology). Regarding the tumor-specificity of thalidomide derivatives, GSPT1 degradation triggers the integrated stress response pathway in tumor cells, resulting in apoptosis [20, 21]. Furthermore, Sellar et al. [22] reported that cells with higher levels of translation, including cancer cells [23], are more susceptible to the effects of GSPT1 degradation; by this means, normal cells would be relatively protected from thalidomide derivatives targeting GSPT1.

We previously demonstrated that GSPT1 depletion by siRNA induced cell-cycle delay in primary astrocytes [6]. In the present study, we showed that GSPT1-KO delayed the growth of U87 glioblastoma cells. Induced cell death and delayed cell growth are important factors in the context of treatment strategies for malignant tumors [24, 25]. The present study demonstrated that GSPT1-KO U87 glioblastoma cells and CC-885 treated U87 glioblastoma cells enhanced apoptosis at both cellular and individual levels. Taken together, it can be concluded that drugs which decrease GSPT1 protein levels are promising candidates for glioblastoma therapy.

Few studies have examined the relationship between GSPT1 expression and the prognosis of patients with cancer. Long et al. showed that GSPT1 is a prognostic biomarker and promoter of malignant colon cancer [26]. GSPT1 protein was upregulated in colon cancer, and GSPT1 expression positively correlated with tumor size. However, they did not examine the relationship between GSPT1 expression and patient prognosis. Bioinformatics analysis has indicated that *GSPT1* mRNA expression is upregulated in liver cancer and that patients with high expression of *GSPT1* mRNA have poor prognosis [13]. The group proposed that GSPT1 promotes tumor growth and invasion in liver cancer. Wang et al. identified five genes, including *GSPT1*, that may serve as potential prognostic biomarkers and therapeutic targets in triple-negative breast cancer [27]. It has been reported that GSPT1 is negatively regulated by miR-27b-3p via combining with the 3′-untranslated region and that patients with gastric cancers and lower miR-27b-3p expression demonstrate a poorer prognosis [28].

Although we demonstrated that a severe decrease in GSPT1 expression was induced by CC-885 and that GSPT1-KO in U87 glioblastoma cells resulted in prolonged survival in the mouse model of transplanted brain tumors, *GSPT1* mRNA expression levels in 87 human glioblastoma samples from Kobe University Hospital showed no significant relationship with patient prognosis. Our clinical study was consistent with the TCGA data of both Glioblastoma 2013 and 2008 (Fig. 6D and https://www.cbioportal.org/). However, TCGA datasets of liver cancer have shown that high *GSPT1* mRNA expression is associated with poor prognosis (https://www.proteinatlas.org/ENSG00000103342-GSPT1/pathology). Conversely, TCGA datasets of colorectal and renal cancers reportedly showed high *GSPT1* mRNA expression and better prognosis. Moreover, TCGA data showed no relationship between *GSPT1* mRNA levels and prognosis in patients with stomach or lung cancers, wherein GSPT1 has been reported to be a tumor-promoting factor [14, 15]. There are several possible reasons for this discrepancy of GSPT1 involvement. One possibility is a discrepancy between *GSPT1* mRNA and GSPT1 protein expression levels. GSPT1 may not affect cell growth or malignancy if a certain amount of protein is present in the glioblastoma cells. Furthermore, the expression levels of therapeutic target genes do not always correlate with prognosis. It has been recognized that patients with high expression levels of hormone receptors in breast cancer have a better prognosis [29]. In glioblastoma, EGFR aberration is a major genetic abnormality, and high EGFR expression is associated with angiogenesis and invasion; nonetheless, patients with high EGFR expression do not always have a significantly worse prognosis than those with low EGFR expression [30, 31]. Taken together, it can be deduced that the relationship of GSPT1 expression levels with malignancy and prognosis varies depending on the tumor type and even the tumor subtype; therefore, further studies exploring these findings are required in the future.

Although CC-885 bridges CRBN to GSPT1 as a neosubstrate of the CRL4^CRBN^ ubiquitin ligase complex, leading to the degeneration of GSPT1, CC-885 triggers the degradation of additional CRBN substrates [10, 11], such as Ikaros, Ailos, cyclin-dependent kinase 4 (CDK4) [32], and bcl2-interacting protein 3 like (BNIP3L) [33], potentially triggering unexpected off-target effects. Degradation of CDK4 and BNIP3L have been associated with cell-cycle delay [34] and decreased apoptosis [35], respectively. Accordingly, GSPT1-selective immunomodulatory drugs [10], such as CC-90009, MRT-2359, and SJ6986 [20, 21, 36], have been developed, and CC-90009 and MRT-2359 are currently being explored in clinical trials involving patients with acute myeloid leukemia and lung cancer, respectively. Nevertheless, since GSPT1 is involved in the synthesis of many proteins as a translational terminator, targeted GSPT1 depletion/deletion may have unexpected effects. Although we previously identified RAC1-GSPT1 signaling in the cell-growth pathway [6], further studies are required to identify the downstream target of GSPT1 that regulates cell growth and death without unexpected effects.

In conclusion, we demonstrated that GSPT1 is a novel target for the development of therapeutics against glioblastoma. Although GSPT1-selective immunomodulatory drugs and other drugs/molecules that decrease GSPT1 levels via pathways other than the CRL4^CBRN^ pathway may be promising candidates for glioblastoma and other types of tumors, GSPT1 possesses a potential weak point, and can cause unexpected consequences via its functions as a translational terminator. Therefore, identification and elucidation of the downstream target of GSPT1 is required to develop novel drugs with minimum off-target effects on the antitumor RAC1-GSPT1 signaling axis.

## Materials and methods

### Chemicals and antibodies

Staurosporine was purchased from Cayman Chemical (Ann Arbor, MI, USA). Antibodies for GSPT1 (Atlas Antibodies, Cat# HPA052488, RRID:AB_2681849), cleaved PARP1 [Cell Signaling Technology, Danvers, MA, USA; PARP (Asp214) (D64E10) XP, Cat# 5625, RRID:AB_10699459], and cleaved caspase-3 [Cell Signaling Technology; cleaved caspase-3 (Asp175) (5A1E), Cat# 9664, RRID:AB_2070042] were purchased. A magnetic-agarose conjugated monoclonal antibody for HA (Cat# M180-10) and HRP-conjugated monoclonal antibodies for HA (Cat# M180-7; RRID:AB_11124961), FLAG (Cat# M185-7; RRID:AB_2687978), GAPDH (Cat# M171-7, RRID:AB_10699462), α-tubulin (Cat# PM054-7, RRID:AB_10695326), and β-actin (Cat# PM053-7, RRID:AB_10697035) were obtained from MBL International (Nagoya, Japan).

### Plasmids

Human *GSPT1* in pEGFP-C1 (Takara Bio, Kusatsu, Japan), named EGFP-GSPT1, has been described previously [6]. Human *GSPT1* was cloned into 3×FLAG-CMV-14 (Sigma-Aldrich, St. Louis, MO, USA), pEGFP-N3 (Takara Bio), and pcDNA3.1 (Thermo Fisher Scientific, Waltham, MA, USA) plasmids, and named GSPT1-FLAG, GSPT1-EGFP, and GSPT1, respectively. N-terminally HA-tagged ubiquitin (Ub) plasmid has been described previously [37].

### Cells

Primary astrocyte cultures were prepared from the cerebral cortex of postnatal day 2 (P2) *GFAP–Cre*^*–/–*^*;RAC1* ^*flox/flox*^*;tdTomato* (WT) and *GFAP–Cre*^*+/–*^*;RAC1* ^*flox/flox*^*;tdTomato* (RAC1-KO) mice, as described previously [6]. Dissected cerebral cortices were dissociated in EMEM supplemented with 10% FBS, 100 units/mL penicillin, and 100 μg/mL streptomycin, and cultured in 25 cm^2^ flasks (2 brains per flask) (Corning Inc., Corning, NY, USA). After 5–7 days, the flasks were subjected to continuous shaking for 2 h to obtain purified astrocytes. The percentage of RAC1-KO primary astrocytes obtained from *GFAP–Cre*^*+/–*^*;RAC1* ^*flox/flox*^*;tdTomato* mice with tdTomato fluorescence ranged 80–90%, as described previously [6].

U87 glioblastoma cells were obtained from ATCC (Manassas, VA, USA; RRID:CVCL_0022), and U87 cells expressing iRFP720 were kindly provided by Mischel PS (Department of Pathology, Stanford University). GSPT1-KO U87 cell lines were established using a genome-editing method. Briefly, TrueGuide synthetic sgRNA for GSPT1 (GGCGGTGCAACCACAGACTT, ID: CRISPER721175, Thermo Fisher Scientific) and TrueCut Cas9 protein (Thermo Fisher Scientific) were electroporated into U87 cells using the Neon transfection system (Thermo Fisher Scientific). After confirming genome editing using the GeneArt Genome Cleavage Detection Kit (Thermo Fisher Scientific), genome-edited single U87 cells were plated in 96-well plates and proliferated. After proliferation, GSPT1-KO clones, a heterozygous GSPT1 KO (named A-2) and a homozygous GSPT1 KO (named A-7) were selected by immunoblotting using a GSPT1 antibody. To establish GSPT1-KO U87 clones with stable GSPT1-FLAG expression, *GSPT1-FLAG* plasmid was transfected into the GSPT1-KO U87 clone (A-7) and then selected in the presence of 300 μg/mL G418. Establishment of the clone (Rescued GSPT1-KO) was confirmed by immunoblotting using a GSPT1 antibody. All U87 cells (lines and clones) were maintained in D-MEM (FUJIFILM, Osaka, Japan) containing 10% FBS (Nichirei Biosciences, Tokyo, Japan), 100 units/mL penicillin, and 100 μg/mL streptomycin in a 5% CO_2_ humidified incubator at 37 °C.

### Cell cycle analysis

After 2 h of shaking, purified primary WT and RAC1-KO astrocytes were obtained after trypsinization. The cells were electroporated with *EGFP(C1)* plasmid using a NEPAgene21 electroporator (NEPAGENE, Ichikawa, Japan). For rescue experiments, *EGFP-GSPT1* plasmid was electroporated into RAC1-KO primary astrocytes. Three types of electroporated primary astrocytes (WT astrocytes with EGFP, RAC1-KO astrocytes with EGFP, RAC1-KO astrocytes with EGFP-GSPT1) were cultured in 35-mm glass-bottom dishes (MatTek, Ashland, MA, USA). After 48 h of plating, cells were imaged every 20 min for 72 h at 37 °C in 5% CO_2_ using a computer-assisted incubator fluorescence microscope system (LCV110, Olympus, Japan), as described previously [6]. This system enabled ultra-long-term imaging of living cells without removing them from culture conditions. Cell cycle (doubling time) was defined as the time from one to the next cytokinesis. Astrocytes with EGFP fluorescence were analyzed, and cells with tdTomato were considered to be RAC1-KO astrocytes. Experiments were performed in duplicate, and at least three independent transfection experiments were conducted.

### Growth rate of U87 glioblastoma cells

WT, heterozygous GSPT1-KO (A-2), homozygous GSPT1-KO (A-7), and Rescued GSPT1-KO with GSPT1-FLAG (rescued GSPT1-KO) U87 cells (0.5 × 10^4^ each) were prepared on 12-well plates. Twenty-four, 48, 72, and 96 h after preparation, the cells were trypsinized, and the total number of cells were counted using an automatic cell counter (TC20; Bio Rad Laboratories, Hercules, CA, USA).

### Immunoprecipitation and immunoblotting for detecting GSPT1 ubiquitination by CC885 and apoptosis by staurosporine

GSPT1-FLAG and HA-Ub plasmids were co-transfected into U87 glioblastoma cells using Lipofectamine 3000 (Thermo Fisher Scientific). Thirty hours after transfection, the cells were treated with the indicated concentrations of CC-885 for 16 h. Cells were lysed in homogenizing buffer [38] by sonication in the presence of protease and protein phosphatase inhibitor cocktails (Nacalai Tesque, Kyoto, Japan; Cat# 03969-21), and 1% Triton X-100. Total lysates were centrifuged at 900 × *g* for 5 min at 4 °C, and the protein concentration of the supernatants was determined using a BCA protein assay kit (Thermo Fisher Scientific). Equal amounts of proteins were incubated with 5 μL magnetic-agarose conjugated HA antibodies (MBL) for 2 h at 4 °C. After washing, eluted proteins were subjected to SDS-PAGE, followed by immunoblotting using an HRP-conjugated FLAG antibody and an enhanced chemiluminescence detection system (Clarity; Bio-Rad Laboratories). Comparative loading of HA-tagged Ub and FLAG-tagged GSPT1 was confirmed by immunoblotting using HRP-conjugated antibodies against HA and FLAG.

WT and GSPT1-KO (A-7) U87 cells were seeded into 12-well plates. The cells were treated with an indicated concentration (62.5–500 nM) of staurosporine for 9 h, and then total lysates, after centrifugation at 900 × *g* for 5 min at 4 °C, were subjected to SDS-PAGE. Apoptosis was detected by immunoblotting using cleaved PARP1 antibody. For quantification, the relative expression levels of cleaved PARP1 (between WT and GSPT1-KO U87 cells) were normalized to that of α-tubulin at each treatment pair with the same staurosporine concentration and plotted.

### Transplantation of U87 glioblastoma cells into the brains of nude mice

The following U87 glioblastoma cells were used to produce transplanted brain tumors: WT expressing iRFP720 (WT-RFP), WT, GSPT1-KO, and rescued GSPT1-KO U87 expressing GSPT1-FLAG (Rescued GSPT1-KO). The experiments were performed in three pairs: (1) WT-RFP with DMSO vs. WT-RFP with CC-885 (50 or 100 mg/mL), (2) WT vs. GSPT1-KO, and (3) GSPT1-KO vs. Rescued GSPT1-KO. Randomization was not performed, and no pre-determined exclusion criteria were applied in the present study. Mice from the control group were treated and assessed first, followed by those in the experimental groups.

For the experiments (WT-RFP with DMSO vs. WT-RFP with CC-885), nude mice (8-week-old male BALB/c-nu/nu; CLEA Japan, Tokyo, Japan) were anesthetized and placed in a stereotactic apparatus (Most Versatile Stereotaxic Instrument, Muromachi Kikai Co., Ltd, Tokyo, Japan). A burr hole was drilled 2.0 mm to the right of bregma. A needle was inserted to a depth of 3.5 mm from the surface of the brain, and then 10 μL (1 × 10^5^ cells) of WT-RFP U87 cells were infused. The body weight of the mice was measured twice weekly. The experimental protocol is shown in Fig. 2A. Fifteen days after transplantation (day 15), tumor formation by the transplanted cells was confirmed using an in vivo imaging system (IVIS Lumina LT; PerkinElmer, Waltham, MA, USA). CC-885 dissolved in DMSO (50 or 100 mg/kg) and the same volume of DMSO (control) were intraperitoneally administered to the mice once every alternate day (a total of four times; days 16, 18, 20, and 22), and tumor growth was assessed using IVIS Lumina LT on day 24. To examine the effects of CC-885, mice treated with 100 mg/kg CC-885 were sacrificed two days after the fourth CC-885 treatment under anesthesia. Brains along with the tumors were removed, fixed with 4% paraformaldehyde in 0.1 M phosphate buffer (pH 7.4) for two days, and embedded in paraffin for immunoblotting for GSPT1 and histological evaluation. For the other experiments (WT vs. GSPT1-KO and GSPT1-KO vs. Rescued GSPT1-KO), the experimental protocol without tumor growth assessment using IVIS is shown in Figs. 3C and 4C.

In each group, the survival period (days) from transplantation to death was monitored, and the survival rate was calculated.

### Immunohistochemistry

Serial sections were deparaffinized and immersed in methanol containing 0.3% hydrogen peroxide. After permeabilization with PBS containing 0.3% Tween-20, the sections were heated in 0.01 M citrate buffer (pH 6.0) for 15 min by autoclaving (121 °C, 2 atm). Then, the sections were incubated with primary antibodies (GSPT1, cleaved caspase-3, cleaved PARP1) at 4 °C overnight. Subsequently, the sections were incubated with peroxidase-conjugated anti-rabbit IgG polyclonal antibody (Histofine Simple Stain MAX-PO; Nichirei Biosciences) for 60 min. The reaction products were visualized by immersing the sections in 0.03% diaminobenzidine solution containing 2 mM hydrogen peroxide for 5 min. Nuclei were lightly stained with Mayer’s hematoxylin.

### Measurement of Ki-67 index and positive expression area (%) of immunostaining

Immunohistochemical staining levels were independently evaluated and scored by a neuro-oncologist (K. T.) who was blinded to the experimental data. Ki-67 index was calculated as [(Ki-67 positive cells/total cells) × 100] from five randomly selected fields per section under ×200 middle-power magnification. The positive expression area (% area) of GSPT1, caspase-3, and PARP1 were calculated from three randomly selected fields per section under ×200 middle-power magnification using ImageJ 1.48 software (NIH, Bethesda, MD, USA). Areas containing the tumor alone were analyzed, and areas without tumor cells, including those with necrosis, fibrosis, and blood vessels, were excluded from the analysis. The number of positive pixels was divided by the total number of pixels (negative + positive) in the analyzed area and multiplied by 100 to determine the percentage of positive pixels.

### Patient study (real-time RT-PCR and immunostaining for GSPT1)

Patients with newly diagnosed glioblastoma who were treated and followed up at the Department of Neurosurgery, Kobe University, between January 2007 and July 2016 formed our study population. All 87 patients underwent tumor resection or biopsy and were diagnosed with glioblastoma, followed by radiotherapy and chemotherapy with temozolomide and/or bevacizumab.

Total RNA was obtained from the patient samples (*n* = 87) using a mirVana miRNA Isolation Kit (Thermo Fisher Scientific). Reverse transcription was performed using a High-Capacity cDNA Reverse Transcription Kit (Applied Biosystems) according to the manufacturer’s instructions. Expression levels of *GSPT1* mRNA were assayed quantitatively by the ΔΔ-Ct method of real-time RT-PCR using TaqMan® gene expression assays (Thermo Fisher Scientific). GAPDH was used as the endogenous control. Eighteen paraffin-embedded samples from patients were subjected to GSPT1 immunostaining.

### Statistical analysis

The exact number of experiments for each condition are provided in the figures or figure legends. All data are presented as the mean ± SEM. Two groups were compared using unpaired two-tailed Student’s *t*-test. For comparisons among more than two groups, one-way or two-way ANOVA was performed, followed by Tukey’s post-hoc test for pairwise group differences. Survival was estimated using the Kaplan–Meier method, and significance was determined using the log-rank test. Overall survival (OS) was defined as the time from transplantation to death. Statistical analyses were performed using JMP 11 software (JMP Institute, Cary, NC, USA) or Prism 7.0 software (GraphPad Software, La Jolla, CA, USA), and significant differences were indicated as *P < 0.05, **P < 0.01, ***P < 0.001, and ****P < 0.0001. Sample size calculation was not performed.

### Supplementary information

Supplementary Information: Figures and legends

Original Western blots

## Data Availability

All relevant data are within the manuscript and its supplementary information.
