# Supplementary material for: Potential of GSPT1 as a novel target for glioblastoma therapy
Source: Cell Death Dis. 2024 Aug 8;15(8):572. doi: 10.1038/s41419-024-06967-1 (PMC11310507; doi:10.1038/s41419-024-06967-1)
Supplement: Supplementary file 1 — Supplementary Information: Figures and legends [file 41419_2024_6967_MOESM1_ESM.pdf]

## Supplementary Information: Figures S1–4

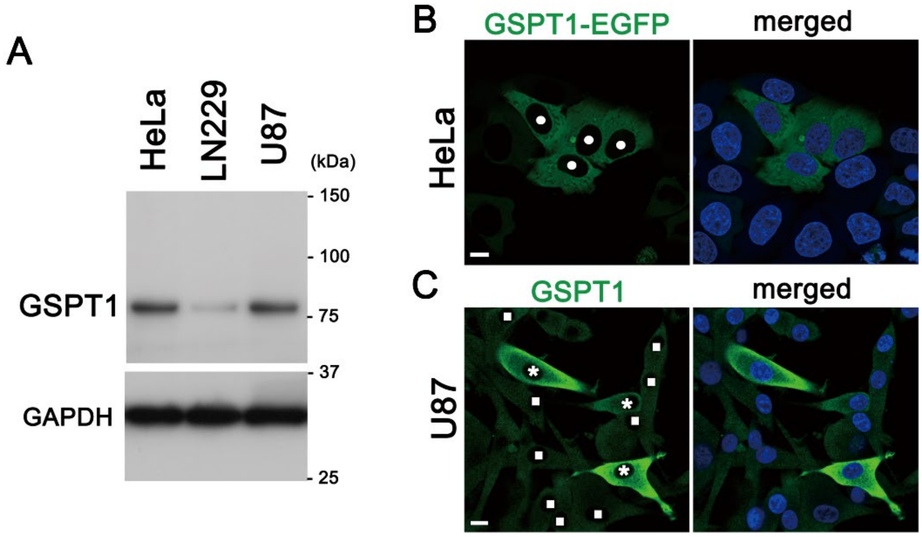


**Supplementary Fig. S1: Expression and cytoplasmic localization of GSPT1.**

**A**, Endogenous expression of GSPT1 was evaluated in HeLa cells along with LN229 and U87 glioblastoma cells by immunoblotting using GSPT1 antibody (HeLa = U87 > LN229). Comparative loading of proteins was confirmed by immunoblotting with an anti-GAPDH antibody. **B**, Cytoplasmic localization of overexpressed GSPT1-EGFP (green; circles) in HeLa cells. **C**, Cytoplasmic localization of overexpressed GSPT1-FLAG (asterisks) was evaluated using GSPT1 antibody in U87 cells. Low expression signal indicated by rectangles may indicate endogenous GSPT1 in U87 cells.

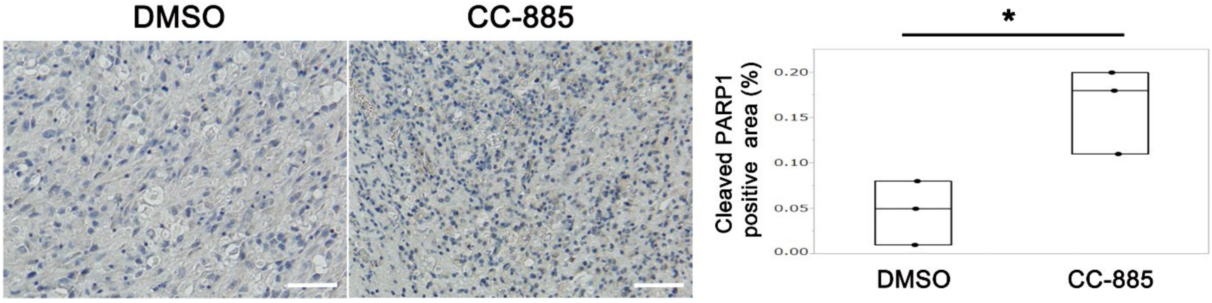


**Supplementary Fig. S2: Vulnerability of GSPT1-KO U87 glioblastoma cells to apoptosis (cleaved PARP1).**

Brain tumors transplanted with WT U87 glioblastoma cells and WT U87 cells treated with CC-885 were removed, fixed, and embedded in paraffin on day 24. Samples were prepared for immunostaining using cleaved PARP antibody. Cleaved PARP1-positive areas were measured using ImageJ software and plotted. n= 3; * $P$ = 0.0265 by Student's *t*-test; Scale bars: 100 µm.

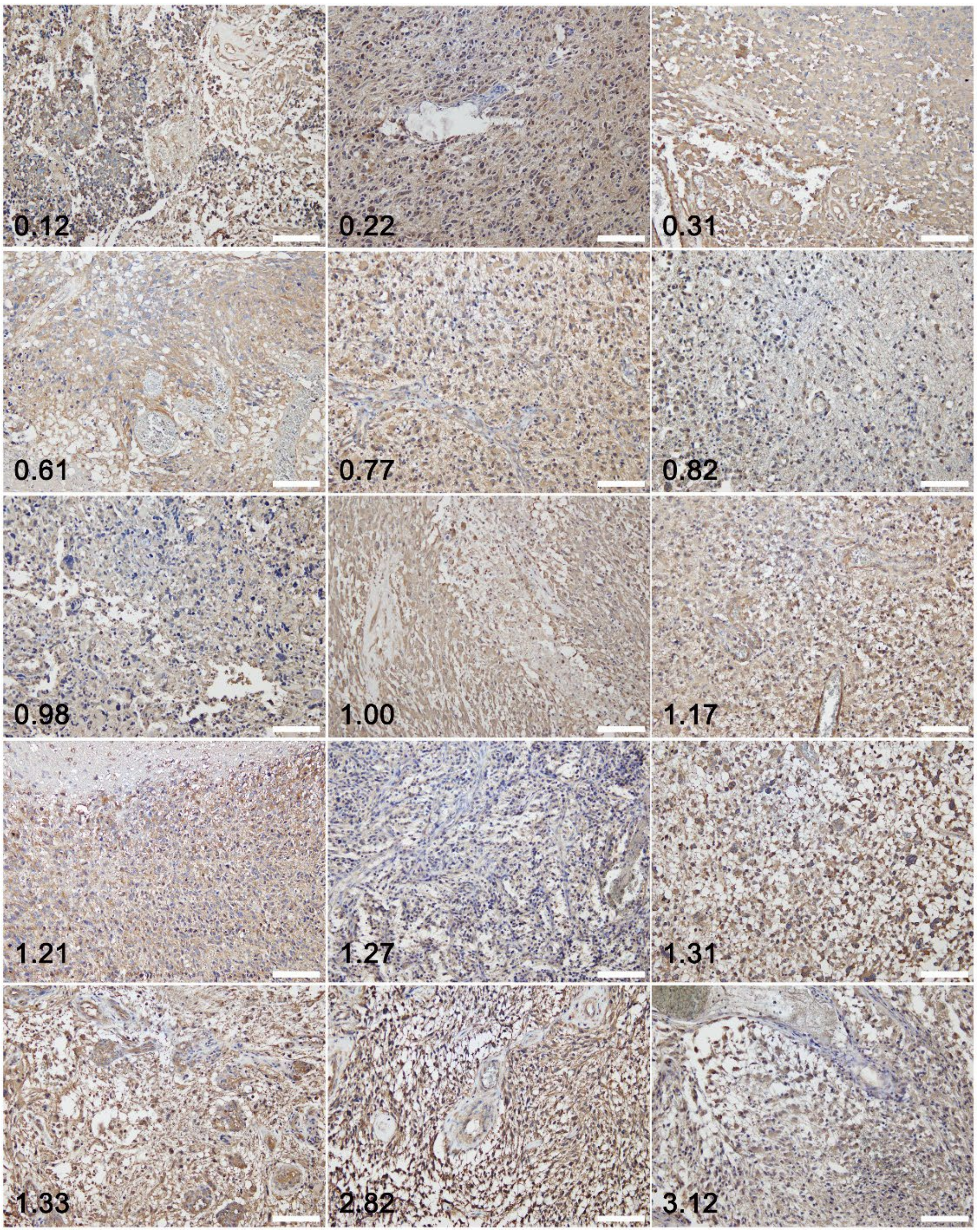


**Supplementary Fig. S3: Immunohistochemistry of GSPT1 in 15 glioblastoma samples.** Fifteen glioblastoma samples were immunostained with GSPT1 antibody. GSPT1 was moderately or strongly expressed in tumor cells in all samples. The numbers at the bottom left of each microphotograph represent the relative expression values of GSPT1 mRNA in real-time RT-PCR analyses. There was no association between GSPT1 immunostaining levels and relative mRNA expression values. Scale bars: 100 μm.

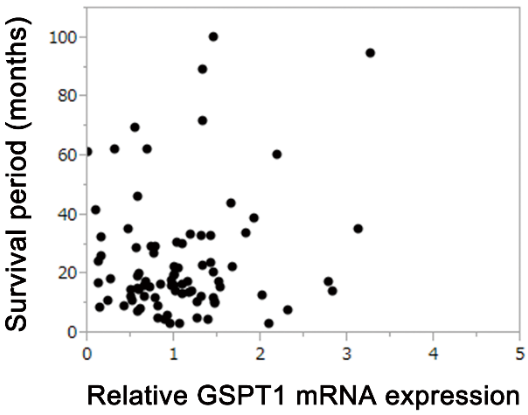


**Supplementary Fig. S4: Scatter plot of GSPT1 mRNA expression level and overall survival time (OS).**

GSPT1 mRNA expression levels in 87 glioblastoma samples were analyzed using real-time RT-PCR. Horizontal axis, relative expression levels of GSPT1 mRNA; vertical axis, overall survival time (months).
