# Supplementary material for: Potential of GSPT1 as a novel target for glioblastoma therapy
Source: Cell Death Dis. 2024 Aug 8;15(8):572. doi: 10.1038/s41419-024-06967-1 (PMC11310507; doi:10.1038/s41419-024-06967-1)
Supplement: Supplementary file 2 — Original Western blots [file 41419_2024_6967_MOESM2_ESM.pdf]

Fig. 1B

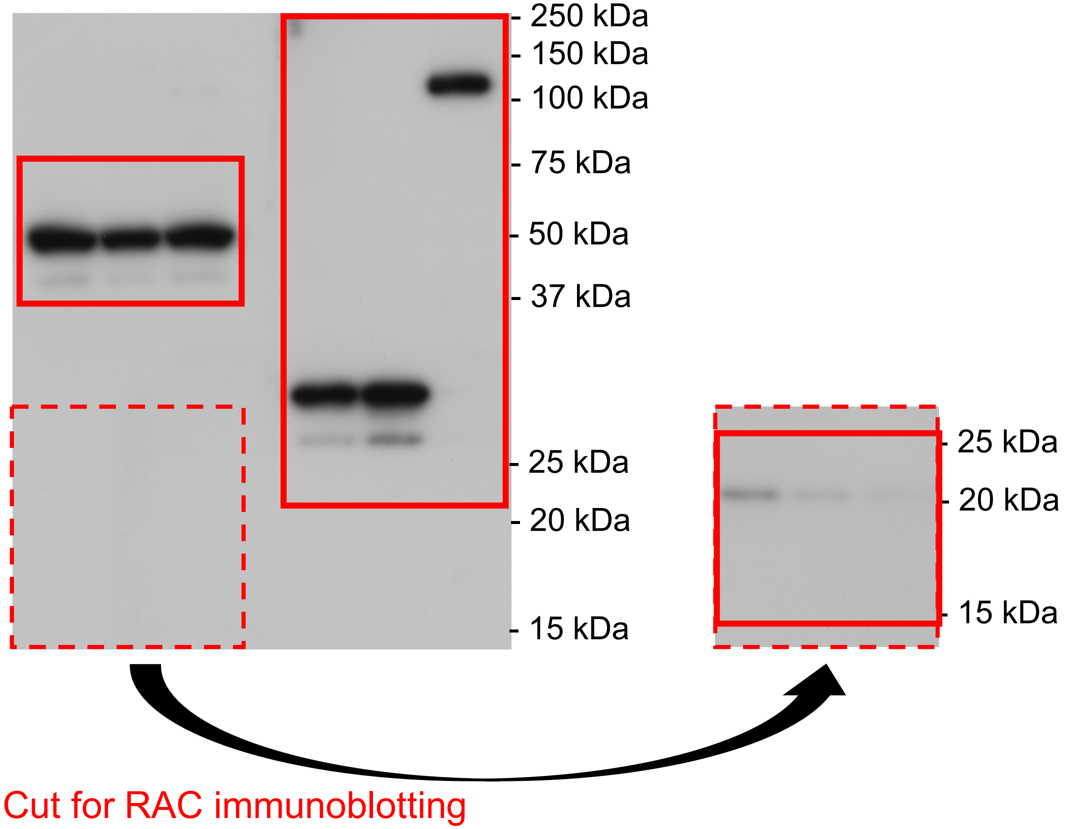

250 kDa
150 kDa
100 kDa
75 kDa
50 kDa
37 kDa
25 kDa
20 kDa
15 kDa
25 kDa
20 kDa
15 kDa
Cut for RAC immunoblotting

Fig. 1C

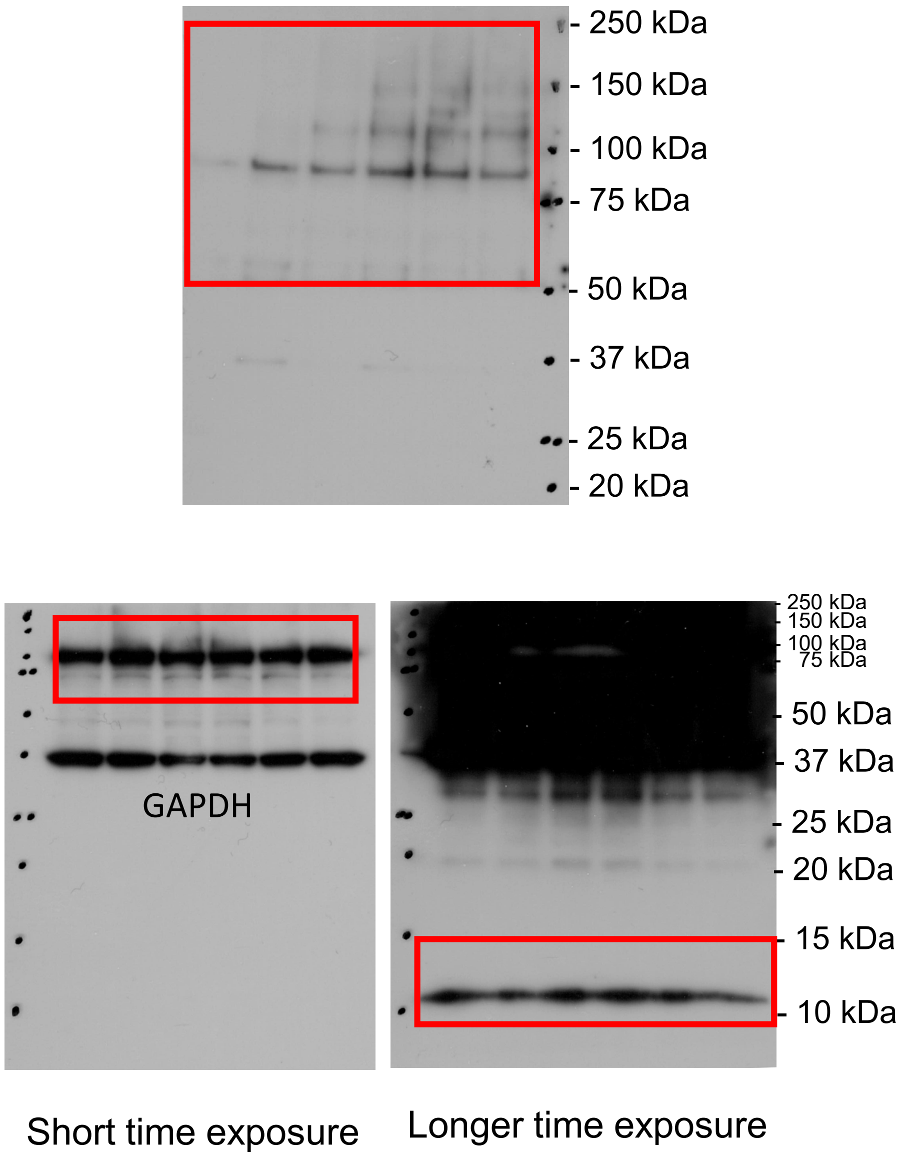

250 kDa
150 kDa
100 kDa
75 kDa
50 kDa
37 kDa
25 kDa
20 kDa
GAPDH
250 kDa
150 kDa
100 kDa
75 kDa
50 kDa
37 kDa
25 kDa
20 kDa
15 kDa
10 kDa
Short time exposure
Longer time exposure

Fig. 2D

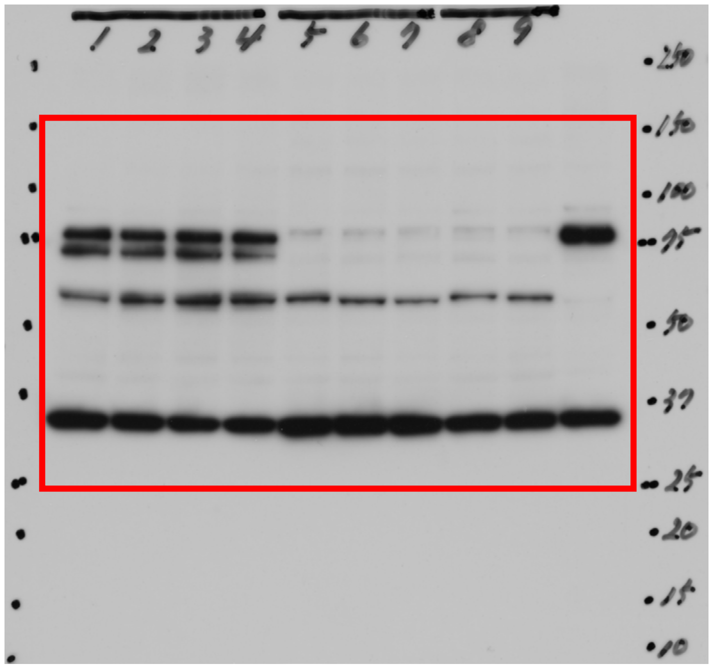

1 2 3 4 5 6 7 8 9
250
150
100
75
50
37
25
20
15
10

Fig. 3A

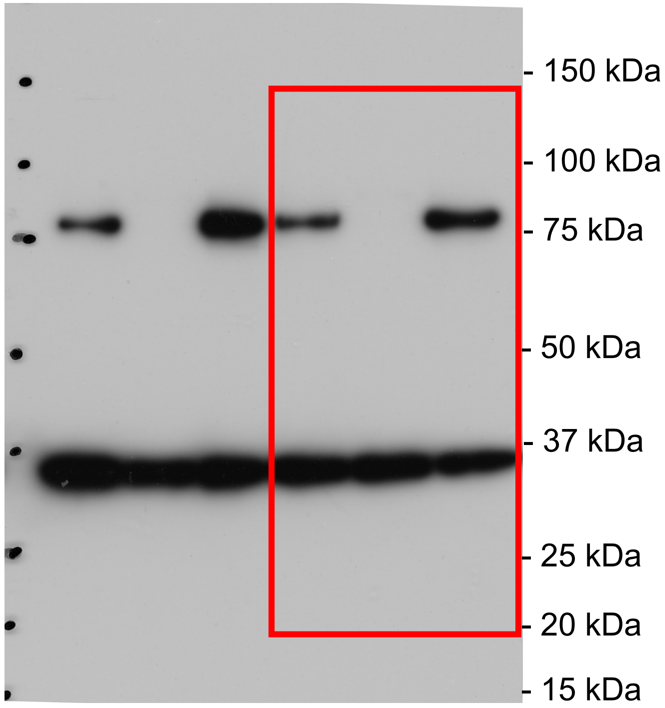

150 kDa
100 kDa
75 kDa
50 kDa
37 kDa
25 kDa
20 kDa
15 kDa

Fig. 4A

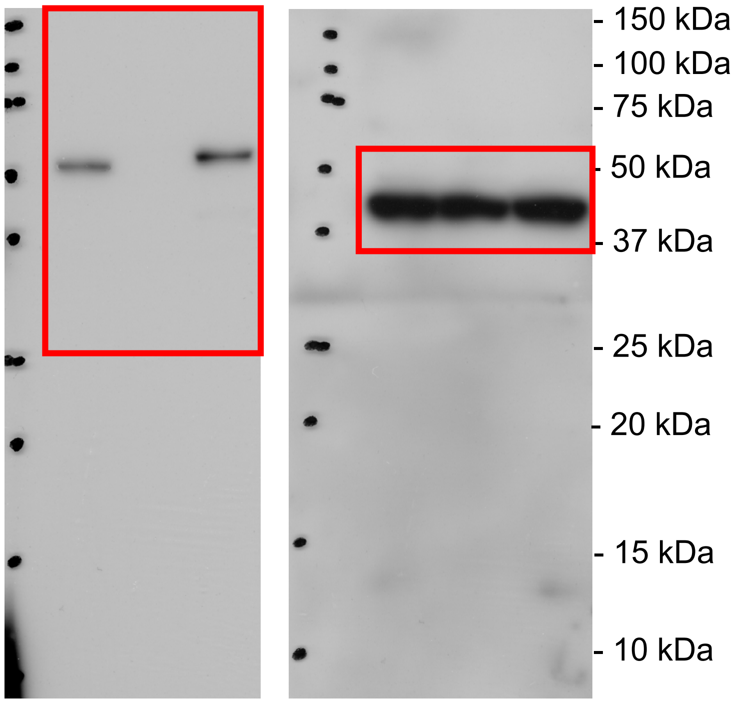
- 150 kDa
- 100 kDa
- 75 kDa
- 50 kDa
- 37 kDa
- 25 kDa
- 20 kDa
- 15 kDa
- 10 kDa

Fig. 5A

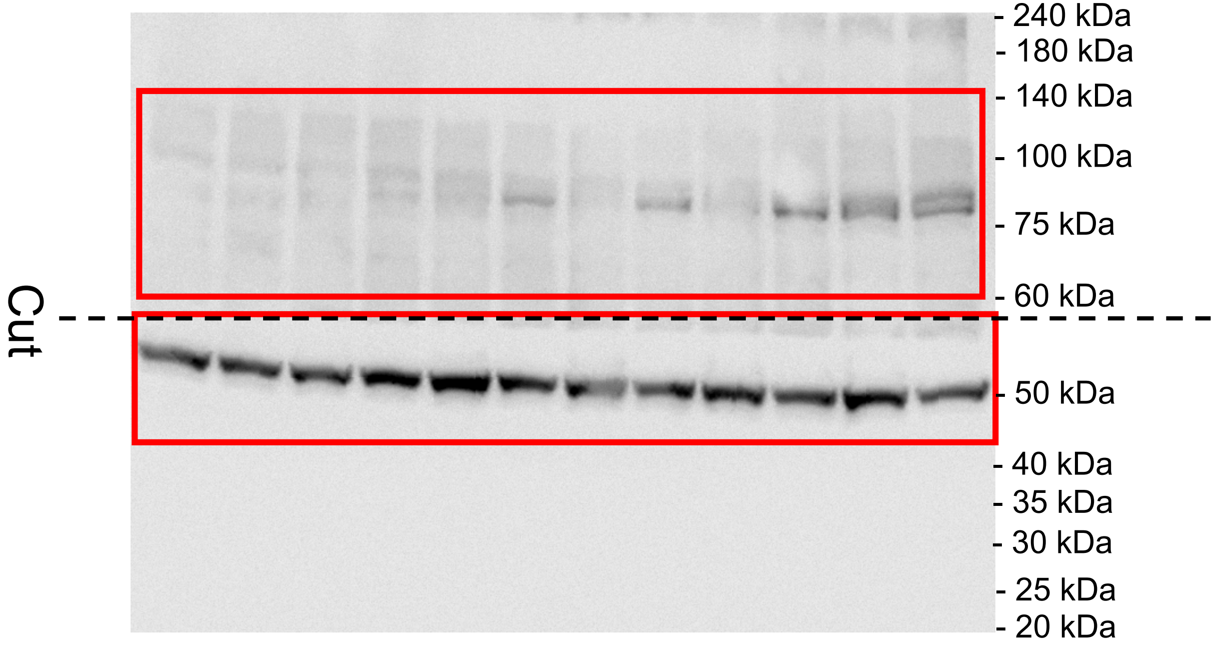
- 240 kDa
- 180 kDa
- 140 kDa
- 100 kDa
- 75 kDa
- 60 kDa
Cut
- 50 kDa
- 40 kDa
- 35 kDa
- 30 kDa
- 25 kDa
- 20 kDa

Fig. S1

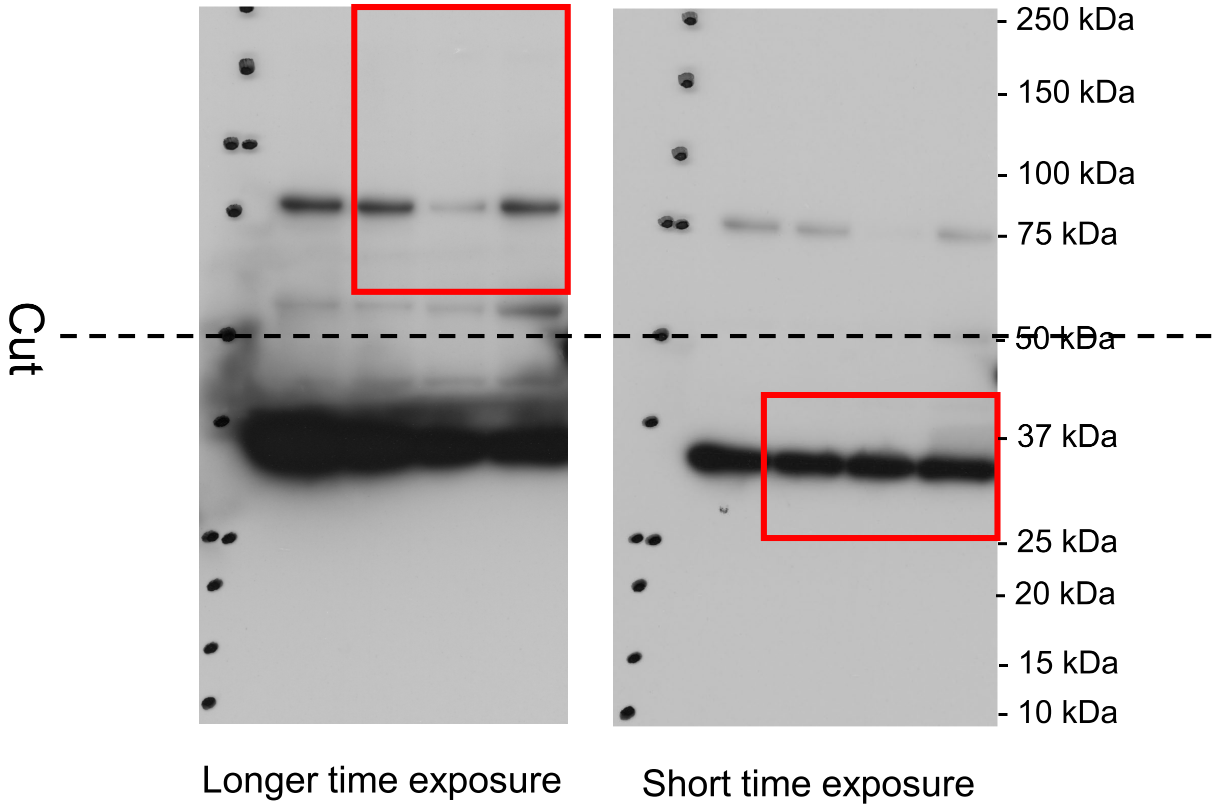
Cut
250 kDa
150 kDa
100 kDa
75 kDa
50 kDa
37 kDa
25 kDa
20 kDa
15 kDa
10 kDa
Longer time exposure
Short time exposure
